# Quality of Sick Child-Care Delivered by Community Health Workers in Tanzania

**DOI:** 10.15171/ijhpm.2018.63

**Published:** 2018-08-15

**Authors:** Colin Baynes, Dominic Mboya, Samuel Likasi, Doroth Maganga, Senga Pemba, Jitihada Baraka, Kate Ramsey, Helen Semu

**Affiliations:** ^1^Mailman School of Public Health, Columbia University, New York City, NY, USA.; ^2^Ifakara Health Institute, Dar es Salaam, Tanzania.; ^3^Tanzania Training Center for International Health, Ifakara, Tanzania.; ^4^Ministry of Health and Social Welfare, Dar es Salaam, Tanzania.

**Keywords:** Child Mortality, Community Case Management, Community Health Workers, Sick Child-Care, Observational Study, Tanzania

## Abstract

**Background:** Community health worker (CHW) interventions to manage childhood illness is a strategy promoted by the global health community which involves training and supporting CHW to assess, classify and treat sick children at home, using an algorithm adapted from the Integrated Management of Childhood Illness (IMCI). To inform CHW policy, the Government of Tanzania launched a program in 2011 to determine if community case management (CCM) of malaria, pneumonia and diarrhea could be implemented by CHW in that country.

**Methods:** This paper reports the results of an observational study on the CCM service delivery quality of a trial cohort of CHW in Tanzania, called WAJA. In 2014, teams of data collectors, employees of the Ministry of Health and Social Welfare trained in IMCI, assessed the IMCI skills rendered by a sample of WAJA on sick children who presented to WAJA with illness signs and symptoms in their communities. The assessment included direct observations of WAJA IMCI episodes and expert re-assessment of the same children seen by WAJA to assess the congruence between the assessment, classification and treatment outcomes of WAJA cases and those from cases conducted by expert re-assessors.

**Results:** In the majority of cases, WAJA correctly assess sick children for CCM-treatable illnesses (malaria, pneumonia, and diarrhea) and general danger signs (90% and 89%, respectively), but too few correctly assess for physical danger signs (39%); on classification in the majority of cases (73%) WAJA correctly classified illness, though more for CCM-treatable illnesses (83%). In majority of cases (78%) WAJA treated children correctly (84% of malaria, 74% pneumonia, and 71% diarrhea cases). Errors were often associated with lapses in health systems support, mainly supervision and logistics.

**Conclusion:** CCM is a feasible strategy for CHW in Tanzania, who, in the majority of cases, implemented the approach as well as IMCI expert re-assessors. Nevertheless, for CCM to be effective, in Tanzania, a strategy to implement it must be coordinated with efforts to strengthen local health systems

## Background

Despite significant declines in the past decade, child mortality in Tanzania remains unacceptably high. Moreover, reduction in child mortality has been uneven across the country, rendering the poorest and least developed regions unlikely to achieve Millennium Development Goal (MDG) 4.^1^ Treatments for preventing deaths of children under-five are well known, simple, and effective, and address the main cases of childhood deaths including malaria, pneumonia and diarrhea.^2^ However, their coverage remains poor in these areas due to health systems constraints, such as supply chain bottlenecks and shortages of trained human resources.^3^

Community health worker (CHW) interventions to manage common childhood illness in the community (ie, community case management or CCM) is a delivery strategy promoted by the global child health community. CCM involves training and supporting CHWs to assess, classify and treat sick children in the home, using an algorithm adapted from the Integrated Management of Childhood Illness (IMCI) approach that focuses on a holistic assessment of the child presenting signs and symptoms using syndromic management, rather than targeting a single disease category. As a strategy for accelerating achievement of goals to reduce childhood mortality, CCM can only succeed if it is delivered at scale and adequately in terms of quality and the strength of the systems that support it. It follows that for countries, such as Tanzania, to adopt and implement CCM effectively, there is a need for knowledge on the degree to which community-based health workers can implement it according to existing clinical standards, the common errors they make in their application of CCM, and the functionality of necessary supervision and logistics systems.^4^

There is evidence on the quality of care delivered through CCM set in limited pilot settings^5,6^ and other studies have examined quality of care of CCM programs being implemented at scale.^7-9^ Some studies have assessed the skills of providers by reviewing service delivery records,^10^ knowledge tests,^11^ role plays and case scenarios,^12^ while others have directly observed CHW services rendered.^13-15^ Research on CCM has compared the effectiveness of CCM programs for a single disease with programs that use CCM for multiple sicknesses, and those which integrate CCM into a broader package of primary healthcare interventions (iCCM).^16^ This study uses rigorous, community-based observation methods with clinical, ‘gold-standard’ re-assessments to evaluate the quality of care for sick children in the context of a pilot program that was implemented from 2010-2015 in three districts of rural Tanzania. Whereas other studies, including those cited above, have shown the effectiveness of CCM in this region, our study aims at demonstrating the feasibility of adapting this approach in Tanzania, which, to date, has not adopted CCM as a strategy to accelerate reduction of childhood mortality. Accordingly, the objectives of this study were three-fold. First, we sought to understand if CHW in Tanzania could implement CCM of major childhood illnesses adequately vis-à-vis national clinical standards. Linked to this, our second objective was to illuminate the clinical pathways toward correct and incorrect management of sick children by CHW in community settings. The third, and last, objective of this study was to contextualize these results in the wider milieu of rural health systems where the pilot program was held. For our third objective, we direct readers’ attention, also, to an earlier publication that draws upon qualitative data from the evaluation of this program and illustrates the perceptions of CHW, clinic-based healthcare workers, district healthcare managers and community members on the feasibility, effectiveness and acceptability of the CCM program.^17^

In 2007, the Government of Tanzania developed the Primary Healthcare Services Development Program (Swahili acronym *MMAM*) which called for a recognized national cadre of CHW.^18^ In 2010, the United Nations Children’s Fund (UNICEF) conducted a situation analysis to clarify optimal ways to operationalize a national CHW program.^19^ Their report recommended that CHW be based in the community, linked to the health system as a salaried and nationally recognized cadre, and formally trained to provide an integrated package of services addressing preventative and curative aspects of primary healthcare, emphasizing maternal, newborn and child health as well as IMCI and management of malaria, pneumonia and diarrhea.

In 2010, the Tanzanian Training Center for International Health (TTCIH), the Ifakara Health Institute (IHI), Columbia University, and the Ministry of Health and Social Welfare launched The *Connect* Project, which operationalized a CHW program based on the above recommendations, in three rural districts.^20^ *Connect* is designed as randomized controlled implementation trial that examines the effect of introducing CHW (called *Wawezashaji wa Afya ya Jamii or* WAJA in Swahili) on maternal and child health and survival in three rural districts over 5 years. *Connect* is registered with the International Standard Randomized Controlled Trial Register number (ISRCTN96819844). This paper explains the implementation and results of an observational study of the quality of sick child-care provided by WAJA, using CCM, within the *Connect* intervention areas. How WAJA performed vis-à-vis national standards, their specific clinical errors, and the systems factors that shape the quality of care, as well as the implications of these for CHW policy in Tanzania, are reviewed and discussed.

## Methods

### Study Setting

This study occurred between June and August 2014 in three rural districts, Kilombero, Rufijii, and Ulanga, which were the intervention districts for the *Connect* Project. Of these villages, 101 were included within the *Connect* trial, and 50 were randomized to receive the WAJA intervention, with the remaining 51 villages serving as controls with a total of 370 000 people under observation in the trial, half exposed to the WAJA intervention. This study took place only in WAJA intervention villages.

### Study Subjects

A total of 142 WAJA were recruited and trained in three cohorts between October 2010 and July 2013. Of these 60 were included in this study, approximately 20 from Rufiji, which comprises of one-third of the study area, and 40 from Kilombero and Ulanga districts. All WAJA were selected based on community requirements and the minimum eligibility criteria outlined for civil service employment in Tanzania. This includes (1) ‘form-four’ education level (equivalent to 10th grade educational-level in the United States); (2) residency in the community they will serve; and (3) selection by their communities. Recruitment processes were led by district leadership, namely District Educational Officers to verify applicants’ eligibility and members of Council Health Management Teams (CHMT), in partnership with leaders of intervention communities, who posted and screened applications, interviewed those qualified and selected finalists to stand for village election. Those WAJA elected by their communities underwent an academic year (9 months) of training at the TTCIH, which developed a CHW curriculum that incorporated the biological, clinical and community aspects of primary healthcare. Core competencies included in the training were health promotion and preventive services including HIV/STI prevention and counseling, education on family planning and distribution of oral contraceptives and condoms, safe motherhood promotion, and aspects of focused antenatal care. As well basic community treatment protocols were included that covered essential newborn care, IMCI, and CCM for malaria, pneumonia and diarrhea. In line with the protocol for civil service employment, for the duration of the project all WAJA were salaried employees of their respective CHMTs.

The CCM component of the training occupied seven sessions and involved both classroom training and clinical practice. CCM implementation was phased into the field operation during the first month of their deployment in order to ensure the presence of supervisors to monitor and coach WAJA to provide CCM in their village, monitoring the standard of care that had been established during training. The content of the WHO/UNICEF, *Caring for the sick child in the community* package for children aged 2 months to 5 years, was adapted by the MOHSW (known in Swahili as *Bangu Kitita*) for use by CHW, such as WAJA, to perform IMCI. WAJA were specifically trained to assess and classify children presenting uncomplicated cases of fever (as a proxy for malaria), a cough with fast breathing (as a proxy for pneumonia), diarrhea, eye infections, anemia, measles, malnutrition, and ear infections, as well as check their immunization status, assess for general and physical danger signs, and refer them as appropriate. Treatment functions of the WAJA, in accordance with CCM practice, were to treat children classified as having malaria, pneumonia and diarrhea with artemisinin combination therapies (ACT), antibiotics and oral rehydration salts (ORS) with zinc. Box 1 provides definitions of correct assessment and illness classifications and information on how WAJA should respond to children presenting with symptoms CCM-treatable illnesses. The ACT used for first line anti-malarial treatment is called Artemether Lumefantrine (Alu). During the first year of the program, the MOHSW changed the first line antibiotic for pneumonia from cotrimoxazole to amoxicillin. Also in line with adaptions to the national standard of care, in 2012, the project districts rolled out rapid diagnostic malarial test (m-RDT) kits for assessing febrile illness to health facilities and WAJA.

For this study, WAJA were observed in their case management of children aged 2 months to 5 years who presented with a new complaint during the study enrollment period. Eligible children must have been 2 months or older, and have had a complaint not previously addressed by the WAJA. Children with a condition deemed by the expert observer to require immediate referral were also excluded from the study. Criteria for exclusion and immediate referral included symptoms that would prevent the WAJA from giving the sick child oral medications. In our study, these were convulsions, unusual sleepiness or unconsciousness, inability to drink or eat anything or vomiting everything. Children excluded from eligibility on account of their age or because they had a complaint previously assessed by WAJA still helped by WAJA; however, data from these encounters were not gathered for the study. Children excluded because they required immediately referral received assistance from the study team to ensure that they reached a healthcare facility and received timely care. For data collection, WAJA were asked to provide routine IMCI visits every day over a period of a week during which the research teams made unannounced visits to WAJA in their community settings.

### Data Collection

Data was collected first by direct observation, aided by a case management observation tool, of WAJA sick child care by trained data collectors with backgrounds as IMCI-trained clinicians (either a ‘master IMCI trainer’ or district IMCI Coordinator). Direct observation and conduct of WAJA performance was conducted in two steps. First, a data collector directly observed WAJA conducting a consultation with the patient, recording implementation of key screening, assessment, classification, treatment and counseling functions (see Supplementary file 1). Second, another data collector who was not present during the initial examination, re-assessed the same child independently using national IMCI guidelines, recording findings on their screening, assessment, classification, treatment and counseling of the sick child using a comparable checklist as used during the direct observation before (see Supplementary file 2). Data collectors then conducted exit interviews to assess caregivers’ knowledge of the child’s condition and treatment instructions, as well as their perceptions of the quality of WAJA performance (see Supplementary file 3). WAJA did not participate in the exit interview or re-assessment of the sick child. Data collectors finally interviewed WAJA using a structured questionnaire on the availability of essential supplies, supervision from the district and village working conditions (see Supplementary file 4). Data collectors also inspected drug stocks and examined WAJA client registers to estimate service utilization.

Five teams collected data, each comprised of four data collectors each (all IMCI-trained and experienced Medical Officers), with one data collector assigned as a team supervisor. The teams participated in an initial week-long training on the assessment protocol, methodology and research ethics. Data collectors practiced direct observation, re-assessment and WAJA and caregiver interviews in a classroom setting using role-plays, and in clinical practice and community settings in a section of Kilombero district not used in the assessment. During the assessment, teams from the *Connect Project* assessed the quality of data collection and provided mentoring and routine troubleshooting to ensure reliability of results captured.

### Study Design and Sample Size

This was a cross-sectional study of the quality of WAJA sick child-care using CCM. Our aim was to enroll a representative sample of WAJA vis-a-vis the geographic spread of the study area, population distribution, and the demographic characteristics of the WAJA themselves. The overall sampling frame (n = 142) included all WAJA that had completed training between 2010 and 2013. The selection of WAJA was stratified by district, as indicated above, because WAJA deployment was concentrated more heavily in Kilombero and Ulanga district (the larger two study sites) where two-thirds of WAJA resided, whereas only one-third resided in Rufiji district. Accordingly, 40 WAJA from the former districts and 20 from the later district were selected respectively, for a total sample size of 60. In addition to the region where they were deployed, the 60 WAJA were selected to be representative of the entire group in terms of their gender, age, and training cohort. Based on WAJA supervision reports from the month prior to the assessment (May 2014) it was expected that WAJA would see an average of five sick children over the course of a week, with each WAJA observed for a week in total. Thus the actual sample size, with an anticipated total of 300 sick child observations, was estimated to detect a +/- 6.2% precision in our point estimates of the proportion of sick children assessed, classified and treated correctly by WAJA for all illness classifications, estimated at 50% (precision being higher for proportions that are farther from the mean, ie, 15% of 75%). In this, we assume 95% confidence and a design effect of 1.2 to account for intra-class correlation of observations among children seen by the same WAJA.

Sampled WAJA were asked to provide routine IMCI visits everyday over a period of a week during which the research teams made unannounced visits to observe them in their respective communities. Of all the children whom WAJA encountered during this study, 43 (12%) were not enrolled because they did not meet the eligibility standards described above. Eight other cases (2%) were removed from the study after the data was collected if research managers could not attest to its reliability after data reconciliation steps, which involved data collectors and supervisors. Within the week spent with each sampled WAJA, the research team was able to conduct five valid, usable sick child observations per WAJA.

### Outcomes

Quality of care was defined using indicators of correct case management based on existing consensus indicators for the quality of sick childcare developed for IMCI by the World Health Organization (WHO).^21^ These were then adapted to reflect the Tanzania clinical guidelines to assess the quality of IMCI in facilities, and again, to ensure relevance for the community settings in which WAJA work. Box 1 describes the definitions of correct assessment, CCM-treatable illness and danger signs. The primary outcomes of interest were children checked for fever, cough, fast breathing and diarrhea, children assessed for general and physical danger signs, children whose classification from WAJA matched that of the expert re-assessor, children with CCM-treatable classifications whose classification from WAJA matched that of the expert re-assessor, children with CCM-treatable classifications whose treatment from WAJA matched that of the expert re-assessor.

**Box 1. Definitions of Correct Assessment and Illness Classifications**

**Definition of correct assessment for CCM-treatable illness**

*
Assessment of uncomplicated fever:* WAJA takes the sick child’s temperature with a thermometer and asks the caregiver about the presence or history of fever, or the caregiver offers this information; WAJA tests sick child’s for presence of malarial parasites using rapid diagnostic tests (m-RDT).

*
Assessment of cough with fast breathing:* WAJA asks caregiver about the presence of cough or caregiver provides this information; WAJA asks about the number of days of the cough; WAJA counts the sick child’s respiratory rate using a timing device (eg, timer or watch). Assessment of diarrhea: WAJA asks caregiver about the presence of diarrhea or caregiver provides this information; WAJA asks caregiver about the number of days with diarrhea and the presence of blood in stool.

**
Definition of CCM-treatable illnesses (ie, classification based on assessment)
**

*
Uncomplicated fever:* Child with fever lasting less than 7 days and a negative response to m-RDT, but without any other danger signs. WAJA treats with paracetamol.

*Uncomplicated malaria:* Child with fever lasting less than 7 days and a positive response to m-RDT, but without any other danger signs. WAJA treats with ACT (Alu).^a^

*
Uncomplicated cough with fast breathing:* Child with cough for less than 21 days and with fast breathing (respiratory rate of 50 breaths per minute or more for ages 2-12 months and 40 breaths per minute for ages 12 months to 5 years). WAJA treats with amoxicillin.

*Uncomplicated diarrhea:* Child with diarrhea for less than 14 days without blood in stool and no danger signs. WAJA treats with ORS and zinc.

**Definition of danger signs requiring referral**

*
General danger signs:* Child is unable to drink; feed or breast feed vomits everything; has or had convulsions; lethargy or unconsciousness.

*
Physical danger signs:* Child has chest in-drawing; palmar pallor; stunting or wasting as per Middle-Upper Arm Circumference tape; swelling of both feet (bipedal oedema).

^a^ In cases where m-RDT were not available, then malaria is classified presumptively on the basis of presence of childhood fever for less than 7 days.

Secondary outcomes included rational use of antibiotics and ACT, tabulating the frequency of WAJA dispensing medication to children *without* suspected malaria or pneumonia. Measures of health system support reflect the frequency of supervision visits made to the WAJA during the quarter prior to their enrollment in the assessment, the availability of essential drugs and supplies to WAJA, and use of service delivery data for planning at the village level.

### Data Analysis

Data were entered into Stata 13 (College Station, TX), with data processing and reconciliation taking place in Dar es Salaam, Tanzania and New York, USA. Key indicators were calculated as proportions and with 95% CI, adjusted for clustering of sick child consultations performed by the same WAJA. The inter-rater agreement for key variables, that is the proportion of classification and treatment recommended by IMCI expert re-assessors that were also enacted by WAJA, is also reported as a kappa statistic with 95% confidence intervals. A scale proposed by Landis and Koch^22^ was used to interpret the magnitude of agreement for a range of kappa values as follows: Fair: *K* = 0.21-0.40, Moderate: *K* = 0.41-0.60, Substantial: *K* = 0.61-0.80, Almost perfect: *K* = 0.81-1.00. We performed a ‘simplified clinical pathways analysis’ in order to identify the proportion of WAJA errors at each step of the CCM algorithm (assessment, classification, and treatment) for consultations where a child was given a classification from the expert IMCI re-assessor.

## Results

### WAJA Characteristics

As explained above, this assessment took place as an embedded study within *Connect*, a randomized controlled implementation trial of the impact of WAJA deployment on child health and mortality. Impact results of *Connect* are being prepared for dissemination in a separate publication. Table 1 presents the characteristics of the WAJA included in the sample. WAJA were predominantly male (63%), and the median age was 28 with a range from 23 to 41. As per the sampling methodology, the WAJA representatively reflect the relative size of their respective training cohorts, with 40% coming from cohort 1 and 2, trained in 2010-2011 and 2011-2012, respectively, and 20% coming from the third cohort, trained in 2012-2013. Client registers from the three months prior to the assessment were available for review by data collectors, who excluded five WAJA registers because the information in them seemed unrealistic and could not be explained by WAJA. The median number of sick children aged two months to 5 years seen by these WAJA (n = 55) during this period was 66, with median ranging between 44 and 100.

**Table 1:** Characteristics of WAJA Included in the Sample (n = 60)

**Characteristics**	**No. (%)**
Gender	
Male	38 (63)
Female	22 (37)
Age	
20-25	7 (12)
26-30	27 (45)
31-35	25 (42)
36-40	1 (2)
Family status	
Married	31 (51)
No children	22 (36)
1 child	18 (30)
2 children	8 (13)
3 or more children	12 (20)
Time since deployment as WAJA	
Three years (deployed in 2011)	24 (40)
Two years (deployed in 2012)	24 (40)
One year (deployed in 2013)	12 (20)
Educational attainment	
‘Form-four’ (US 10^th^ grade education)	60 (100)
Connect WAJA training program	60 (100)
Median number of sick child visits in previous quarter	
(March-June 2014) *[IQR]*	66 *[44-100]*

Abbreviations: WAJA, *Wawezashaji wa Afya ya Jamii*; IQR, interquartile range.

### Patient Characteristics

A total of 300 sick child consultations were observed in the study (Table 2). Though additional sick children were provided care during the study, these were ultimately removed from the analysis because they were aged less than two months or over 5 years, did not present a new complaint to WAJA or required immediate referral. Roughly a third of the children observed 1-year old or younger (34%), a quarter between one and two years old (26%), and less than a quarter were between three and five years of age (22%). More than half of the children observed were male (54%). The vast majority of children observed were accompanied by a female caregiver (97%). The most common complaints as reported by the caregivers to the WAJA were cough (63%), fever (51%) and diarrhea (14%). About three-fourths (76%) of caregivers reported more than one complaint, and the median number of complaints was two.

**Table 2:** Characteristics of Sick Children and Their Caregivers (n = 300)

**Characteristics**	**No. (%)**
Child’s age (mon)	
3-12	101 (34)
13-24	78 (26)
25-36	57 (19)
37-48	41 (14)
49-60	23 (8)
Child gender	
Female	138 (46)
Male	162 (54)
Gender of caregiver	
Female	292 (97)
Male	8 (3)
Presenting complaint of sick children included in the study as reported by caregiver to WAJA	
Cough	190 (63)
Fever	153 (51)
Diarrhea	42 (14)
Other problems mentioned^a^	55 (18)
Difficulty breathing	6 (2)
Vomiting	9 (3)
Red Eye	2 (1)

Abbreviation: WAJA, *Wawezashaji wa Afya ya Jamii.* ^a^Abdominal pain (14), cold/runny nose (14), flu (13), skin rash (4), ear problem (2), mouth sores (1), general weakness/headache (7).

### Case Mix

Table 3 shows the case mix of sick children seeking care for a new episode of illness from the WAJA on the day of the assessment visit, as defined by the IMCI-trained medical professional who re-assessed the children. The majority of the sick children presented with at least one CCM-treatable illnesses (29% with malaria, 26% with cough and difficulty breathing and 17% with diarrhea). Roughly one-fifth of the sick children observed presented with at least one danger sign (19%), or an uncomplicated fever (19%). The most common danger signs were palmar pallor, diarrhea with blood in stool, fever for more than seven days, and swelling of both feet and ear infections.

**Table 3:** Proportion and Number of Observed Clinical Interactions Where IMCI-Trained Medical Professional Determined Signs/Symptoms to Be Present (n = 300)^a^

**Characteristics**	**No. (%)**
CCM-treatable illness	
Malaria (Fever for <7 days, positive on m-RDT)	86 (29)
Cough with fast breathing	77 (26)
Diarrhea (<14 days and no blood in stool)	51 (17)
No CCM-treatable condition	68 (23)
One or more danger signs	56 (19)
Danger signs
Palmar pallor (anemia)	37 (12)
Diarrhea with blood in stool	5 (2)
Fever for ≥7 days	3 (1)
Swelling of both feet	3 (1)
Ear infection	3 (1)
Diarrhea for more than 2 weeks	1 (0.33)
Pus draining from eye	1 (0.33)
Clouding of cornea	1 (0.33)
Vomits everything	1 (0.33)
Extreme lethargy, unconscious	1 (0.33)
Chest in-drawing	0 (0)
Unable to eat or drink	0 (0)
Convulsions	0 (0)
Severe under-nutrition (red on MUAC tape)	0 (0)
Other signs/classifications	
Cough	220 (44)
Uncomplicated, non-malaria fever (<7 days)	58 (19)
Behind on immunizations/vitamin A	15 (5)
Moderate malnutrition (yellow on MUAC tape)	15 (5)

Abbreviations: CCM, community case management; m-RDT, rapid diagnostic malarial test; MUAC, mid-upper arm circumference. ^a^Signs and symptoms are not mutually exclusive; one child can have more than one classification.

### Quality of Assessment and Treatment

The key results related to the quality of sick child-care in the areas of assessment, classification and treatment are presented in Table 4. In column 1, we present indicator tasks that were taken from CCM guideline used to train and assess WAJA. In column 2, we present the number of children for whom the task should have been performed based on the re-assessment of each sick child that was carried out by the IMCI trained clinician. In column 3, we present the proportion of the sick child consultations in which the WAJA carried out the same task (ie, Column 1) that the Expert Re-assessor carried out (ie, Column 2). In column 4, the 95% CI for each estimate. The proportions shared in column 3, thus represents the proportion of cases in which the WAJA carried out the sick child consultation the same way as the Expert Re-assessor, as per each indicator task. We use this measure as a proxy for the level of correctness with which WAJA conducted care for sick children. It can be inferred that the proportion that remains after the proportion reported in column 3 is deducted from 100% is, indeed, the proportion of cases, per task, in which the necessary task was performed by the Expert Re-assessor only.

**Table 4:** WAJA Performance - Proportions of Children for Whom Specific Case Management Tasks, Necessary According to IMCI Expert Re-assessors, Were Performed by WAJA

**Indicator (Task)**	**N** ^a^	**Percent** ^b^	**95% CI**
Assessment			
Children checked for fever, cough and diarrhea	300	90	86.0%–93.2%
Children with cough assessed for presence of fast breathing through counting respiratory rates	220	91	87.8%–95.4%
Children assessed for 4 general danger signs	300	89	84.9%–92.3%
Children assessed for 4 physical danger signs	300	39	33.4%–44.8%
Classification			
Children whose classification given by WAJA matches all the classifications given by IMCI-trained medical professional evaluator (all IMCI classifications, including ‘no illness’)	300	73	67.6%–77.9%
Children whose classification of CCM-treatable illness(uncomplicated fever or malaria, cough with fast breathing or diarrhea, and ‘no CCM-treatable illness’) given by WAJA matches those given by IMCI-trained medical professional evaluator	300	83	71.8%–81.6%
Treatment of child illness			
Children with one or more CCM-treatable illnesses (uncomplicated fever or malaria, cough with fast breathing or diarrhea) who are correctly prescribed medications for their illness and receive correct first dose from WAJA	232	78	73.7%–83.7%
Children with uncomplicated malaria who are prescribed an ACT correctly and receive correct first dose from WAJA^c^	86	84	74.2%–90.1%
Children with uncomplicated fever, no malaria, who are given paracetamol correctly and receive correct first dose from WAJA^d^	58	84	72.4%–92.7%
Children with uncomplicated cough and fast breathing who are prescribed an antibiotic correctly and receive correct first dose from WAJA	77	74	62.8%–83.3%
Children with uncomplicated diarrhea who are prescribed ORS and zinc correctly first dose from WAJA	51	71	56.2%–82.3%
Rational use of medicines			
Children without a CCM-treatable classification that WAJA prescribes an ACT, antibiotic or ORS/zinc	68	35	8.3%–27.1%
Referral for danger signs			
Children with general and/or physical danger signs needing referral who are referred	56	66	52.2%–78.2%
Counseling			
Children prescribed one or more treatment (ACT, antibiotic and/ or ORS), who caregivers received dose, duration and frequency counseling messages about administering treatment^e^	203	74	78.5%–88.5%
Children prescribed one or more treatment (ACT, antibiotic and/ or ORS), whose caregiver was able to describe correctly how to give the treatment^f^	203	67	69.9%–81.4%
Children whose vaccination status is checked	246	88	77.6%–87.3%
Children with uncomplicated diarrhea whose caregivers are advised to give extra fluids and continue feeding	51	69	65.8%–91.4%

Abbreviations: WAJA, *Wawezashaji wa Afya ya Jamii*; IMCI, Integrated Management of Childhood Illness; CCM, community case management; ACT, artemisinin combination therapies; ORS, oral rehydration salts. ^a^Child consultations in which the task in Column 1 was performed by the Expert Re-Assessor and should have been performed by WAJA. ^b^% of the child consultations for which the task that was performed by the Expert Re-Assessor and should have been performed by WAJA (ie, Column 1) was performed by WAJA. ^c^Includes those who were classified as having fever for less than 7 days without danger signs, positive results on m-RDT; excludes children with fever for more than 7 days to whom m-RDT was not administered. ^d^ Includes only those who tested negative for malaria using m-RDT. ^e^ Among those prescribed a CCM medication, irrespective of classification status. ^f^ Among those without any danger signs requiring referral; of those that did not have vaccination status checked 19 did not have a card available at time of visit.

Notably, WAJA correctly assessed 90% of children for the presence of febrile, respiratory and diarrheal illnesses. Among the children with cough, 91% were correctly assessed for the presence of fast breathing by counting respiratory rate. Although 89% of children were correctly assessed for four general danger signs, a much lower proportion of them were correctly assessed for physical danger signs (39%). The physical danger signs most overlooked were malnutrition assessment using MUAC tape (assessed for 54% of children) swelling in both feet of the sick child (assessed for 55%), and chest in-drawing (assessed for 65%).

The illness classifications assigned by WAJA were consistent with those of the IMCI-trained medical professional who conducted the re-assessments for 73% of the sick children observed. The proportion rose to 83% when considering only classification of CCM treatable illnesses. Over three-fourths (78%) of the 232 children with a CCM-treatable illness, including uncomplicated fever and malaria diagnosed through use of rapid diagnostic tests, cough and fast breathing as a proxy for pneumonia, and diarrhea, respectively, received the correct treatment from the WAJA for their illness(es). A breakdown by type of illness shows that children with a febrile condition were more likely to receive correct treatment (84%). Out of the 86 children classified with malaria, 84% (72) received ACT correctly, while of the 58 children who were classified as having uncomplicated fever 84% (49) also received the recommended dose of paracetamol from WAJA. Out of the 162 children presenting with a fever, 18 (6%) were not administered an m-RDT because of stock-outs among the WAJA from whom they sought care (see Table 5). They, accordingly, were not classified as CCM-treatable for a febrile condition, and referred to a clinic.

**Table 5:** Inter-rater Agreement of Classification and Treatment Performance Between WAJA and IMCI Expert Re-assessor

**Indicator**	**Agreement With IMCI Expert Re-assessor**	**Kappa Value [95% CI]**
Children whose classification given by WAJA matches all the classifications given by IMCI-trained medical professional evaluator (all IMCI classifications, including ‘no illness’)	73%	0.72 [0.69-0.75]
Children whose classification of CCM-treatable illness (uncomplicated fever or malaria, cough with fast breathing or diarrhea, and ‘no CCM-treatable illness’) given by WAJA matches those given by IMCI-trained medical professional evaluator	83%	0.82** [0.80-0.84]
Children whose classification of uncomplicated malaria or fever by WAJA matches those given by IMCI-trained medical professional evaluator	89%	0.88** [0.86-0.90]
Children whose classification of cough and fast breathing by WAJA matches those given by IMCI-trained medical professional evaluator	81%	0.81** [0.78-0.84]
Children whose classification of diarrhea by WAJA matches those given by IMCI-trained medical professional evaluator	78%	0.77* [0.74-0.80]
Children whose treatment received from WAJA for uncomplicated malaria matches those recommended by IMCI-trained professional evaluator	84%	0.84** [0.82-0.86]
Children whose treatment received from WAJA for cough and fast breathing matches those recommended by IMCI-trained professional evaluator	74%	0.74* [0.71-0.77]
Children whose treatment received from WAJA for diarrhea matches those recommended by IMCI-trained professional evaluator	71%	0.71* [0.68-0.74]

Abbreviations: WAJA, *Wawezashaji wa Afya ya Jamii*; IMCI, Integrated Management of Childhood Illness; CCM, community case management. ** ‘Almost perfect’ agreement (*K* = 0.81-1.00). * Substantial agreement (*K* = 0.61-0.80).

A total of 77 children enrolled in the assessment with persistent cough and fast breathing, of which 74% received correct antibiotic treatment from WAJA. The assessment identified 51 children with CCM-treatable forms of diarrhea, of which 36 (71%) received the correct regimen of ORS and zinc from the WAJA. Out of the five children with diarrhea that presented with bloody stool, three continued to receive treatment from WAJA, against protocols for CCM. Of note, of the 51 sick children with a CCM-treatable classification that did not receive correct treatment from WAJA, 36 (71%) were infants less than 12-months of age. 35% (24) of the 68 children not classified with an illness, despite not needing treatment as specified in clinical guidelines, still received either an ACT, antibiotic, ORS or zinc from the WAJA. Two-thirds (37) of the 56 children presenting with danger signs received a referral from the WAJA.

Table 5 presents the kappa values, which reflect the probability that inter-rater agreement presented in Table 4 can be ascribed to subject properties, ie, comparable adherence to the IMCI algorithm, or chance. Kappa values were calculated to assess the level of inter-agreement between WAJA and IMCI expert re-assessor for IMCI illness classification (all, CCM-related and specifically for uncomplicated malaria and fever, cough and fast breathing and diarrhea), treatment provision and instruction (uncomplicated malaria, cough and fast breathing and diarrhea). Kappa values ranged from 0.71–0.88 (mean = 0.79), and six out of the eight values reflected ‘almost perfect’ between WAJA and IMCI expert or sick children’s caregiver (See Table 5).

### Quality of Counseling to Caregivers

Of the 203 caregivers whose children, sick with a CCM-treatable illness, received treatment from WAJA, 151 (74%) received information about the dose, frequency and duration of treatment; however, only 137 (67%) of the caregivers of sick children were able to correctly describe how to administer the treatment (Table 4). Of the 246 children who did not require a referral owing to presentation of danger signs, WAJA checked the vaccination status of 217 (88%). WAJA advised 35 out of the 51 caregivers of children with CCM-treatable diarrhea (69%) to give extra fluids and continue feeding their sick child.

### Health Systems Supports to WAJA

For the 3 months prior to the assessment (March–May, 2014), on average, each WAJA had two IMCI-related supervision exchanges with their health facility supervisor (Table 5). Two-thirds of WAJA report to routinely participate in village government meetings and collaborate with village governments, using their service delivery information to plan village health activities. About 85% of WAJA involved in the assessment had all the critical CCM drugs (ACT, amoxicillin, ORS and zinc, and paracetamol). Information on availability of key IMCI supplies is also provided. 74% of WAJA enrolled had MUAC tape available during the assessment, and 86% had stock of malaria rapid diagnostic tests. See Table 6, which provides information on essential health systems supports that were available to WAJA during the time of the assessment.

**Table 6:** Health System Supports for CCM, as Reported by Sampled WAJA

**Supervision**	
Average number of IMCI-related supervisions from health facility supervisor in the past quarter *[IQR]*	2 *[0, 6]*
Average number of IMCI-related supervisions from village supervisor in the past quarter *[IQR]*	1 *[0, 6]*
Proportion of WAJA who participate in village government meetings, including use of their health management information for planning	67%
**Drug supply (WAJA had in stock on the day of the visit)**	
All critical CCM drugs (ACT, amoxicillin, ORS, zinc, paracetamol)	79%
ORS	94%
Zinc	79%
ORS and zinc	79%
ACT	95%
Amoxicillin	90%
**Supplies**	
MUAC tape	74%
IMCI job aid (*Bangu Kitita)*	93%
Malaria rapid diagnostic kit	86%
Timing device (timer or cell phone with timer on phone)	100%
Thermometer	85%

Abbreviations: WAJA, *Wawezashaji wa Afya ya Jamii*; IMCI, Integrated Management of Childhood Illness; CCM, community case management; ACT, artemisinin combination therapies; ORS, oral rehydration salts; MUAC, mid-upper arm circumference; IQR, interquartile range.

### Clinical Errors Pathways

**Figure 1:**
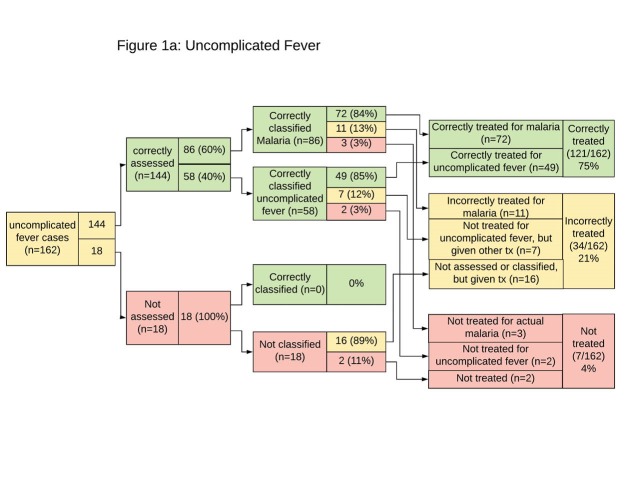
Uncomplicated Fever Clinical Errors Pathway.

**Figure 2:**
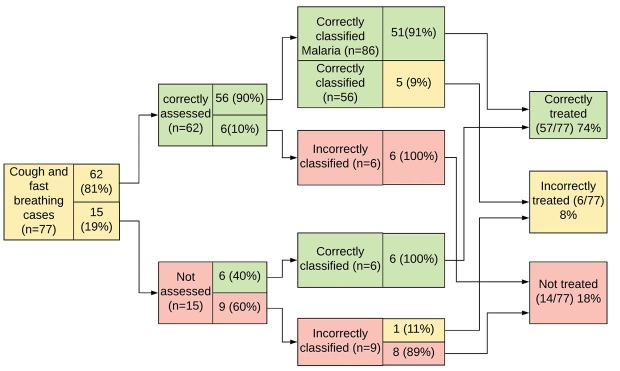
Cough and Fast Breathing Clinical Errors Pathway.

**Figure 3:**
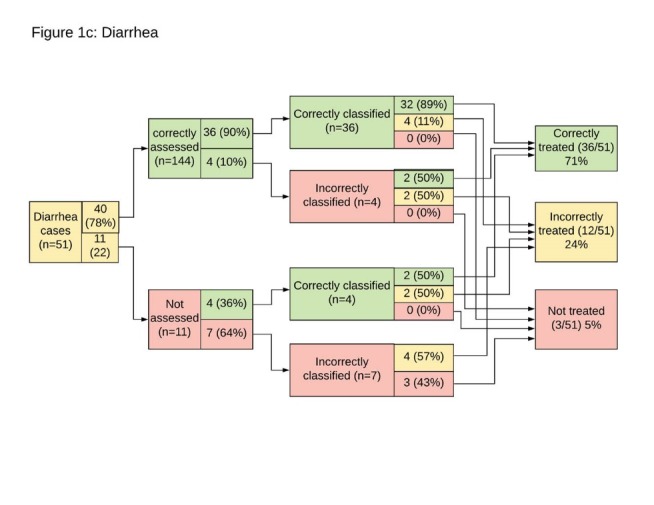
Diarrhea Clinical Errors Pathway.

Figures 1-3 presents an analysis of the clinical steps followed by WAJA during these episodes and pointing out their common errors. Availability of rapid diagnostic kits for testing malaria enabled the correct assessment and classification of most children presenting with fever (144/162). Of these children, 86 were correctly classified with malaria and 58 with uncomplicated fever. Of the 86 with malaria, 14 were incorrectly treated, 3 because of the WAJA were stocked out of ACT. The other 11 also presented with other CCM-treatable conditions and received other treatments. Of the 58 children testing negative for malaria and classified with uncomplicated fever, 49 received correct treatment from WAJA (paracetamol and counseling to the caregiver). Of the 162 children presenting with fever, 18 (11%) could not be tested for malaria because WAJA were stocked out of malaria rapid diagnostic kits.

A total of 77 children were classified with cough and fast breathing. Of these, 62 (81%) were correctly assessed. Mistakes (n = 15) occurred because WAJA failed to ask about time since onset of coughing, check for chest in-drawing, and count breaths. Of the 62 children assessed correctly, 6 were mistakenly classified as not having cough or fast breathing. These children also presented with cases of diarrhea, malaria, or both, together with their cough and rapid breathing. Of the 62 children with cough and fast breathing who were classified correctly, 57 went on to receive the correct antibiotic treatment for suspected pneumonia. Five who were assessed and classified correctly also presented with malaria and diarrhea, and received treatment for those illnesses, but not for suspected pneumonia. One child that was assessed and classified incorrectly also presented with another CCM-treatable condition and was treated for it, but, again, not for suspected pneumonia. 14 children who presented with cough and rapid breathing received no treatment for any illness, six of whom were classified correctly and eight incorrectly.

Fifty-one children presented with diarrhea, of whom 40 were correctly and 11 were erroneously assessed by WAJA. Five of those mis-assessed had also been classified with suspected pneumonia or malaria, and 6 were mis-assessed because WAJA failed to assess thoroughly for dehydration (observe drink and abdomen skin folding back). Errors in classification of children with diarrhea (n = 11), were all due to mis-classifying their dehydration status. In total, 36 children received the correct treatment of ORS and zinc for diarrhea, while 12 were incorrectly treated, receiving either ORS or zinc, but not both. Three of the 51 children presenting with diarrhea were mis-assessed, mis-classified and incorrectly treated. They also presented with malaria. Of the cases in which ORS was provided without the zinc accompaniment (n = 9), six of the occurrences were amongst WAJA stocked out of zinc.

## Discussion

Over 30 countries have adopted CCM as a strategy to increase access to and coverage of treatment interventions for childhood illness.^23^ This is, to the best of our knowledge, the first study to assess the quality of CCM in Tanzania. *The Connect Project* carried out this intervention with the objective of demonstrating that community residents, having received the level of training and implementation support described in this paper, could provide care for sick children, comparably to their facility-based counterparts who, at the time of this study, were the only cadres authorized to do so. In our evaluation of this proposition, we found 78% of children with a confirmed CCM-treatable classification were treated correctly per CCM guidelines. This compares favorably with results from health facilities evaluated for quality of care for sick children during the roll out of facility-based IMCI in Tanzania, where it was found that 74% of children presenting with the same illnesses received correct treatment.^24^ This study demonstrated that WAJA correctly assessed 90% and correctly classified 73% of the sick children enrolled, as compared to results from IMCI assessments at facilities, which found that 90% and 63% of cases encountered by facility providers were correctly assessed and classified.^25^ To assess the influence of location and years of deployment on WAJA performance, we analyzed the prevalence of errors separately according these two variables (region where WAJA were deployed and years since completion of training and deployment). With this, we found no appreciable differences in error levels among WAJA deployed to different regions and WAJA that completed training in different cohorts. That this study employed re-examination of sick children by a medical professional trained in IMCI is a particular strength, and allows us to provide information on the quality of the care for specific, confirmed CCM classifications. Another strength of this study is that it was community-based, in contrast to other studies, which are facility-based use patient data, we observed WAJA in the villages where they ordinarily work.

Acute respiratory infections (ARIs) are the most common illnesses in childhood, and pneumonia is responsible for 15% childhood mortality in sub-Saharan Africa^26^ and Tanzania, specifically.^27^ According to the Demographic and Health Survey for Tanzania, published in 2016, roughly 40% of children with ARI receive appropriate treatment.^28^ This study and others reinforce that further effort is needed to improve its identification and treatment at the facility and community-level. New diagnostic technologies for pneumonia may be useful,^29^ if they are affordable and embedded in functional logistics systems. Though our findings may question the assertion that CHW can rationally dispense antibiotics – 35% of children not presenting with a CCM-treatable illness received treatment from WAJA – it bears note that 15 of those cases were of children who received ACTs from WAJA stocked out of m-RDT. However, the results presented question if m-RDT are sufficient to improve case management of febrile illness. Even among malaria cases identified with m-RDT, 84% received the correct ACT treatment from WAJA. This is slightly less than the 88% of children who were correctly treated for malaria at facilities during facility-based IMCI assessments. Lapses in the availability of m-RDT and implementation of effective supervision suggests that, as in other studies, weak health systems linkages undermine the potential of technologies and expanding the community health workforce to sustain improvements in IMCI.^30^

The fact that a quarter of WAJA observed lacked both ORS and zinc while attending to sick children underlies the gap between ideal and actual correct treatment rates for diarrhea. Further, it underscores the need for strong logistics systems for CCM. However, concerning suspected pneumonia, clinical exam errors were made in the counting of respiratory rates, despite the presence of timing devices, as observed in other assessments of CHW in sub-Saharan Africa.^12^ Over a quarter of basic diarrhea cases identified during the study were incorrectly treated by WAJA. In most of these cases sick children received ORS only, without zinc supplementation. Though related to challenge of stock outs, clinical errors, altogether, underscore the need for strengthening supervision systems and training facility- and community-based providers to correctly manage children presenting with multiple symptoms, recognizing the enhancing effect of zinc on ORS therapy for diarrheal disease and correct use of common CCM tools widely available, such as timers.^31^

This assessment has limitations that should be considered. The CHW and clusters chosen for the assessment are part of a pilot experiment of a program alternative being considered for scale up by the Government of Tanzania. Accordingly, the *Connect Project* provided support to local implementers, at times supporting them to procure and distribute essential medicines and supplies for WAJA when stock outs were inevitable and conduct independent and routine supervision of the WAJA when the means to accomplish this were not available. This, in turn, may have positively biased the results documented in this study. In addition, WAJA may have provided better care than normal because they were being observed, a phenomenon referred to as the Hawthorne effect.^32^ Finally, the exclusion of 43 sick from our study may introduced bias in our analysis. Among those removed, 12 were neonates and six exhibited urgent symptoms that necessitated immediate referral. This decision was consistent with the design of the WAJA service delivery package, which did not permit them to deliver care regimens including intravenous or rectal artesunate and antibiotic treatment, essential for sick neonates and severely ill children over 2 months. Nevertheless, it undermined our study’s potential to discern if WAJA could help those children in the direst need of immediate medical attention. Of those removed, 15 had been attended to by the WAJA for the same condition during the period prior to data collection in their community. The removal of these children from our analysis prevented the study from identifying if errors in the WAJAs’ treatment of these children and if this might explain their need to return to the WAJA for care for the same illness.

The purpose of this study was to inform decisions on the design of a national CHW program in Tanzania where policymakers were deliberating the content of a community-based primary healthcare policy and services package to take to scale. Saliently, WAJA seem to be fulfilling the expectation of bringing child healthcare closer to communities. The subset of WAJA observed with patient registers available saw a median of 66 sick children over the course of a quarter during which WAJA experienced temporary stock outs of essential medicines for CCM and received fewer than the recommended number of supportive supervisions. That *Connect* did not include components to address gaps in the implementation of IMCI at the facilities serving villages where WAJA were deployed, may be problematic for WAJA performance. Evidence from studies conducted in Tanzania shows that many health workers surveyed at facilities are not trained in, able to demonstrate understanding of IMCI,^33^ or do not adhere to IMCI treatment and referral guidelines for children with severe illness.^34^ This study suggests that introducing CHWs in rural Tanzania, through the training and deployment model described in this paper, reaches children with needed services, the quality of which is, in the majority of cases, comparable to that of IMCI experts, when their prognoses are uncomplicated, rather than having children after they have become more severely ill. Roughly one-fifth of children (19%), presented with a danger sign that indicated a severe illness, and two-thirds of these children were referred after receiving pre-referral care from WAJA. Although this proportion may seem low, in some of these communities, referral to a health facility may not be feasible. Furthermore, the referral rates observed are comparable with those of IMCI trained health workers based in facilities in Tanzania and other countries in sub-Saharan Africa.^35,36^ Other findings from the *Connect* evaluation relate to implementation costs and contribute additional evidence pertaining to the feasibility of scaling up the WAJA model. Whereas the annual cost of training Clinical Officers, a three-year training program, was measured at $731 per year, the cost of training WAJA, a one year training program, was $833.50 per year. In terms of the costs of sustaining WAJA after training, the *Connect* cost evaluation found that the per capita cost of deployment and routine management and support of this cadre was $1.16 per capita. This is compared with $2.72, the average annual per capita deployment cost of CHW from programs derived from a meta-analysis of CHW program costs in several countries in sub-Saharan Africa, suggesting that *Connect* cost estimates are an additional favorable feature of the WAJA model.^37^

Specific performance weaknesses observed among WAJA were recognition of physical danger signs, particularly malnutrition, correct assessment and classification of suspected pneumonia, and management of children presenting with multiple illnesses, including effective referrals. Although this is consistent with findings from other settings, poor WAJA performance on danger sign recognition, particularly through use of MUAC tape, is of serious concern as children with these symptoms are at greatest risk. This study underscores the importance of having a functional ‘continuum of care’ that ensures the routine availability of high quality referral-level services at the network of facilities to which CHW will refer sick children. This will prove essential for maintaining effective supervision structures and logistics connections between villages, where CHW will operate, and the formal health system.

## Conclusion

This observation-based assessment of the quality of sick child-care provided by WAJA trained in IMCI and CCM in Tanzania demonstrates the degree to which CHW can perform lifesaving child survival interventions up to nationally accepted standards of quality, as modeled by IMCI-expert re-assessors. The description of the intervention, including the discussion on health systems supports to the WAJA, delineate the processes that generated these encouraging results. Clinical errors emerged prevalently as the WAJA conducted their work, illuminating the effect of a weak health systems context, even in settings where a special project backstops CHW training and management. The Government of Tanzania, as it formulates its national program on CHW, and considers community IMCI and CCM for large-scale implementation, should consider the findings of this study: while training community members to deliver lifesaving interventions to children up to acceptable standards is feasible, it necessitates functionality, responsiveness and support from local health systems. As this study observed, CHW will struggle to achieve their potential unless their clinical skills, particularly those required for managing children with multiple conditions especially pneumonia, and identifying and referring severely ill children, are improved and better supported by reliable supervision and uninterrupted logistics systems.

## Acknowledgments

This work was supported by the Doris Duke Charitable Foundation [grant number DDCF2009058a] and Comic Relief UK [grant number 112259].

## Ethical issues

Permission for this study was accorded by the ethical review boards of the Ifakara Health Institute (IHI/IRB/No. 16-2010), the National Institute for Medical Research’s Medical Research Coordinating Committee (NIMR-CC) (NIMR/HQ/R.8a/Vol.IX/1203) and the Internal Review Board (IRB) of Columbia University Medical Center (Protocol AAAF3452). Research assistants administered formal informed consent procedures and obtained the signature or thumb print of subjects to confirm willingness to participate. Also, this research was guided by the protocol, applicable laws and regulations, and the principles of research ethics as set forth in the Belmont Report. Every care was taken to ensure the privacy and confidentiality of study data.

## Competing interests

Authors declare that they have no competing interests.

## Authors’ contributions

CB designed the study described in this paper, managed data collection, analyzed the data and wrote the paper; DMb, SL, DMa, and JB led field teams in the collection of the data used in the analysis for this paper, reviewed and contributed content to the paper; KR reviewed and contributed content to the paper and raised the funds that supported the conduct of this study; SP and HS reviewed and contributed content to the paper.

## Authors’ affiliations

^1^Mailman School of Public Health, Columbia University, New York City, NY, USA. ^2^Ifakara Health Institute, Dar es Salaam, Tanzania. ^3^Tanzania Training Center for International Health, Ifakara, Tanzania. ^4^Ministry of Health and Social Welfare, Dar es Salaam, Tanzania.

## Key messages

**Implications for policy makers**

- Community case management (CCM) of malaria, pneumonia and diarrhea by community health workers (CHWs) is a feasible strategy for accelerating childhood mortality reduction in settings of rural Tanzania.
- In a large majority of cases, CHW in Tanzania can implement protocols for classifying and treating prominent causes of childhood mortality as well as their Integrated Management of Childhood Illness (IMCI) trained medical professionals.
- For CCM to be successful in Tanzania, and settings like it, coordinated efforts are required that strengthen systems for the extension of essential logistics and supervision from healthcare facilities to communities.

**Implications for public**

The results of this study delineate a feasible strategy for transferring healthcare services that address major causes of childhood deaths from healthcare facilities to communities in resource-constrained settings where children often die because of inadequate access to healthcare. Furthermore, it demonstrates that members of communities, if trained and well managed, in the majority of cases, can perform these services as well as formal healthcare providers that work at static facilities. The results illustrate error pathways in the community health workers’ (CHWs’) assessment, classification and treatment of sick children, which are discussed, later, in context of the wider health system, its functionality and the influence of this on CHW performance. Readers from the public can use this information, reflecting on the situation in their countries and regions, and consider similar approaches for addressing the burden of childhood disease and preventable death where they are from.

## Supplementary files


Supplementary file 1. Sick Child Observation Checklist.

Supplementary file 2. IMCI Expert Reassessment Checklist.

Supplementary file 3. Caretaker Exit Interview.

Supplementary file 4. WAJA Support Questionnaire.
