# Supplementary material for: Quality of Sick Child-Care Delivered by Community Health Workers in Tanzania
Source: Int J Health Policy Manag. 2018 Aug 15;7(12):1097–109. doi: 10.15171/ijhpm.2018.63 (PMC6358652; doi:10.15171/ijhpm.2018.63)
Supplement: Supplementary file 1 — Sick Child Observation Checklist. [file ijhpm-7-1097-s001.pdf]

Connect Project: Quality Assessment of Community Health Agent Service Delivery

Form #1: Direct Observation Checklist of U5 Sickness (child 2 months – 5 years)

Date		District ID		Time Start	
Evaluator ID		CHA ID		Time End	
Child No					
Child Sex	M / F	Caretaker Sex	M / F		
Child Age (months)		Caretaker Relationship to child			

ASSESSMENT MODULE

➔ ***Record what you hear or see.***

A1. What problems does the caretaker state that the child has? Circle all signs mentioned

a.	Fast difficult breathing	(1) Mentioned	(2) Not mentioned
b.	Cough	(1) Mentioned	(2) Not mentioned
c.	Pneumonia	(1) Mentioned	(2) Not mentioned
d.	Diarrhea	(1) Mentioned	(2) Not mentioned
e.	Fever	(1) Mentioned	(2) Not mentioned
f.	Malaria	(1) Mentioned	(2) Not mentioned
g.	Convulsions	(1) Mentioned	(2) Not mentioned
h.	Difficulty drinking or feeding	(1) Mentioned	(2) Not mentioned
i.	Vomiting	(1) Mentioned	(2) Not mentioned
j.	Red eyes	(1) Mentioned	(2) Not mentioned
k.	Any other problems	(1) Mentioned	(2) Not mentioned
		Specify: ___________________________	

Note: if the following have already been mentioned by the caretaker, it is considered a "yes" response.

		Yes	No	Comment
A1	Does the CHA asks if the sick child has had convulsions in the current illness.			
A2<br>A.2.1	Is the child visibly awake (smiling, playing crying with energy?<br>➔ If not visibly awake, does the CHA check for lethargy or unconsciousness (try to wake up the child)?			
A3	Does the CHA checks if the child is convulsing now?.			
A4<br>A4.1	Does the CHA ask if the child has difficulty drinking or feeding?<br>➔ If difficulty drinking or feeding does the cha ask if the child is NOT able to drink or feed ANYTHING?			
A5<br>A5.1	Does the CHA ask if the child is vomiting?<br>➔ If vomiting, did the CHA ask if the child is vomiting EVERYTHING?			
A6<br>A6.1	Does the CHA ask if the child has cough?<br>➔ If cough, did the CHA ask for how long? ____days			
A7<br>A7.1	If cough, does the CHA count breaths in 1 minute?<br>➔ If yes, how many breaths counted? ________b/pm			
A8	Does the CHA look for chest in-drawing?			
A9<br>A9.1	Does the CHA asks about diarrohea.<br>➔ If YES for how long?----------days			
A10	Does the CHA asks if there is blood in stool.			
A11<br>A11.1	Does the CHA ask for fever (reported or now)?			

	➔ If fever, does the CHA ask for how long? ______ days?			
A12<br>A12.1<br><br>A12.2	Does the CHA ask if the child has red eyes?<br>➔ If red eyes, does the CHA ask for how long? ____ days?<br>➔ If has red eyes, does the CHA ask if there generalized body rashes			
A13	Does the CHA asks about ear problems.			
A14	Does the CHA checks for the signs of malnutrition and anemia ?			
A14.1	➔ For a child 6 months to 5 years, does the CHA compare weight for age in road to health card?			
A15	Does the CHA look for swelling signs of wasting?			
A16	Does the CHA look for swelling of both feet?			
A17	Does the CHA look for palmar pallor?			
A18	Does the CHA classify malnutrition and anemia based on findings?			
A19	Does the CHA assess the sick child for immunization status.			
A20	Does the CHA ask if the sick child has other problems.			
A21	CHA correctly classifies the sick child.			
A22	CHA gives correct treatment for IMCI classification.			
A23	CHA demonstrates to mother caretaker how to administer the medicine to the sick child			
A24	Does the CHA explains to mother / caretaker the danger signs for immediate return?			
A25	Does the CHA gives the mother / caretaker a follow up appointment.			
A26	Does the CHA use the service delivery register at any time during the encounter with the child?			
A27<br><br>A27.1	Does the CHA use a malaria rapid diagnostic test at any time during the encounter with the sick child?<br>➔ If used m-RDT, what was the outcome of the test: _____ (positive or negative)			

CLASSIFICATION MODULE

B1. Does the CHA give one or more classifications for the child?

(1) Yes
(2) No → Skip to Treatment Module

➔ ***Note: if the classification is not clear to the observer during the encounter, he/she is permitted to ask the CHA after the encounter is complete what the classification was. Do not ask for each specific classification.***

OBSERVER record all classification below

B2A	One or more danger signs	(1) Yes (2) No
B3A	Diarrhea less than 14 days and no blood in stool.	(1) Yes (2) No
B4A	Diarrhea for 14 days or more	(1) Yes (2) No
B5A	Blood in stool	(1) Yes (2) No
B6A	Fever for last 7 days	(1) Yes (2) No
B7A	Fever for less than 7 days	(1) Yes (2) No

B8A	Convulsions	(1) Yes (2) No
B9A	Not able to drink or eat anything	(1) Yes (2) No
B10A	Vomits everything	(1) Yes (2) No
B11A	Red eye for 5 days or more	(1) Yes (2) No
B12A	Red eye with visual problem	(1) Yes (2) No
B13A	Red eye, less than 4 days	(1) Yes (2) No
B14A	Chest in drawing	(1) Yes (2) No
B15	Fast breathing	(1) Yes (2) No
B16	Very sleepy or unconscious	(1) Yes (2) No
B17	Palmar pallor	(1) Yes (2) No
B18	Compare the weight for age	(1) Yes (2) No
B19	Falls in Red row	(1) Yes (2) No
B20	Fall Green on MUAC tape	(1) Yes (2) No
	Falls in Grey row	(1) Yes (2) No
B21	Swelling of both feet	(1) Yes (2) No
B22	Is the child up to date on vaccines	(1) Yes (2) No
B23	Received appropriate vaccines	(1) Yes (2) No
B24	Other problem, treat at home: Specify: ________________	(1) Yes (2) No
B25	Other problem, refer: Specify: ___________________	(1) Yes (2) No

TREATMENT MODULE

C1. Does the CHA decide to refer to a health facility where required?
(1) Yes
(2) No →*skip to C3.*

C2. Does the caretaker accept referral for the child?
(1) Yes
(2) No, Specify reason: ____

C3. Does the CHA administer or prescribe treatment?
(1) Yes
(2) No →*skip to communication module #D11 if child is referred* ***OR*** *#D5 if child not treated and not referred.*

C4. Circle all treatments given.

	Treatment	Circle	
A	ORS	(1) Yes	(2) No
B	Zinc supplement	(1) Yes	(2) No
C	Paracetamol	(1) Yes	(2) No
D	Cotrimoxazole	(1) Yes	(2) No
E	Amoxycillin	(1) Yes	(2) No
F	Alu	(1) Yes	(2) No
G	Other	(1) Yes	(2) No

C5. Record the following information for each treatment given or prescribed.

Connect Project: Quality Assessment of Community Health Agent Service Delivery

Form #1: Direct Observation Checklist of U5 Sickness (child 2 months – 5 years)

Note: this section is intended to include all treatments, including ORS. If the CHA prescribes home treatment, record the entire treatment. If the CHA treats with one dose for child being referred, record the one dose given, market once for frequency and NA for total days.

A1	Name			B1	Name	
A2	Formulation (i.e. tablet, sachet, liquid)			B2	Formulation	
A3	Amount each time			B3	Amount each time	
A4	Frequency			B4	Frequency	
A5	Total days			B5	Total days	
A1	Name			B1	Name	
A2	Formulation			B2	Formulation	
A3	Amount each time			B3	Amount each time	
A4	Frequency			B4	Frequency	
A5	Total days			B5	Total days	

COMMUNICATION MODULE

- ➔ ***For children being treated at home (or referred because of drug stock-out), start at D1. For children referred, start at D11.***
- ➔ ***D1 to D4 refer to CHA providing the counseling on administration of treatment, whether the treatment is correct or not.***

	ORS	Zinc	Paracetamol	Cotrimoxazole	Amoxicillin	Alu	Other
**D1:** Does the CHA **explain** how to administer treatment(dosage, frequency and duration)	(1) Yes (2) No (3) NA	(1) Yes (2) No (3) NA	(1) Yes (2) No (3) NA	(1) Yes (2) No (3) NA	(1) Yes (2) No (3) NA	(1) Yes (2) No (3) NA	(1) Yes (2) No (3) NA
**D2:** Does the CHA **demonstrate** how to administer the treatment?	(1) Yes (2) No (3) NA	(1) Yes (2) No (3) NA	(1) Yes (2) No (3) NA	(1) Yes (2) No (3) NA	(1) Yes (2) No (3) NA	(1) Yes (2) No (3) NA	(1) Yes (2) No (3) NA
**D3:** Does the CHA **ask the caretaker to repeat back** how to administer the treatment?	(1) Yes (2) No (3) NA	(1) Yes (2) No (3) NA	(1) Yes (2) No (3) NA	(1) Yes (2) No (3) NA	(1) Yes (2) No (3) NA	(1) Yes (2) No (3) NA	(1) Yes (2) No (3) NA

**D4:** Does the CHA **give or ask the mother to give the first dose** of the treatment right away?	(1) Yes<br>(2) No<br>(3) NA	(1) Yes<br>(2) No<br>(3) NA	(1) Yes<br>(2) No<br>(3) NA	(1) Yes<br>(2) No<br>(3) NA	(1) Yes<br>(2) No<br>(3) NA	(1) Yes<br>(2) No<br>(3) NA	(1) Yes<br>(2) No<br>(3) NA

D5. Does the CHA advise on home care?

(1) Yes
(2) No → Skip to question #D7

D6. Circle all advice given by the CHA?

(a) Advised caregiver to give more fluids and continue feeding (1) Yes (2) No
(b) Advised to go to health facility/return if the child cannot drink or feed (1) Yes (2) No
(c) Advised to go to health facility/return if child becomes sicker (1) Yes (2) No
(d) Advised to go to health facility/return if the child has blood in the stool (1) Yes (2) No
(e) Advised caretaker on use of ITN (1) Yes (2) No
(f) Other, specify: ____ (1) Yes (2) No
(g) Other, specify: ____ (1) Yes (2) No
(h) Other, specify: ____ (1) Yes (2) No

D7. Does the CHA ask to see the child's vaccination card?

(1) Yes, and sees card
(2) Yes, but card not available/provided to CHA
(3) No, CHA does not ask

D8. If the vaccination card is NOT available for review, does the CHA ask the caretaker to recall which vaccines her child has received?

(1) Yes
(2) No

D9. Check all advice given by CHA regarding vaccines:

A. Advised to catch up on vaccines, if child is behind (1) Yes (2) No (7) NA
B. Advised to continue getting vaccines according to schedule (1) Yes (2) No (7) NA
C. Advised caretaker where to get vaccines for child (1) Yes (2) No (7) NA
D. Other, specify _____________________________________ (1) Yes (2) No (7) NA

D10. Does the CHA state when s/he will return for a follow up and/or tell caretaker when she should seek the CHA out for follow up?

(1) Yes, and notes it in the register
(2) Yes, but does not note it
(3) No

End of questioning for child treated at home. Continue if child is being referred to a health facility.

D11. For a child being referred, does the CHA explain the need for referral?

(1) Yes
(2) No

D12. For a child being referred, does the CHA do the following:

A. For any sick child who can drink advise to give fluids and continue feeding (1) Yes (2) No (7) NA
B. Advise to keep child warm, if child is NOT hot with fever (1) Yes (2) No (7) NA
C. Write a referral note (1) Yes (2) No (7) NA
D. Arrange transportation (1) Yes (2) No (7) NA
E. Other, specify: ____ (1) Yes (2) No (7) NA

END OF OBSERVATION.
