# Supplementary material for: Quality of Sick Child-Care Delivered by Community Health Workers in Tanzania
Source: Int J Health Policy Manag. 2018 Aug 15;7(12):1097–109. doi: 10.15171/ijhpm.2018.63 (PMC6358652; doi:10.15171/ijhpm.2018.63)
Supplement: Supplementary file 2 — IMCI Expert Reassessment Checklist. [file ijhpm-7-1097-s002.pdf]

Connect Project: Quality Assessment of Community Health Agent (WAJA) Service Delivery

Form 2: Re-examination Checklist for U5 Sickness – child (2 months – 5 years)

Date		District ID		Time Start	
Evaluator ID		CHA ID		Time End	
Child No					
Child Sex	M / F	Caretaker Sex	M / F		
Child Age (months)		Caretaker Relationship to child			

	**Assess** ***(Circle all signs present)***			**Classify**	
A1<br>A2	Does the child have cough?<br>If yes, for how long? ________ days	(1) Yes (2) No	A24	Danger sign:<br>Cough 21days or more?	(1) Yes (2) No
A3<br>A4<br>A5	Does the child have diarrhea?<br>If yes, for how long? ________ days<br>Is there blood in the stool?	(1) Yes (2) No<br><br>(1) Yes (2) No	A25<br>A26<br>A27	Danger sign:<br>Diarrhoea 14 days or more?<br>Blood in stool?<br>Sick but no danger sign:Diarrhoea less that 14 days and no blood in stool?	(1) Yes (2) No<br>(2) Yes (2) No<br>(1) Yes (2) No
A6<br>A7	Is there fever (reported or now)?<br>If yes, for how long? ________ days	(1) Yes (2) No	A28<br>A29	Danger sign:<br>Fever for last 7 days?<br>Sick but no danger sign:<br>Fever for less than 7 days?	(1) Yes (2) No<br>(1) Yes (2) No
A8	Are there convulsions?	(1) Yes (2) No	A30	Danger sign: any convulsions?	(1) Yes (2) No
A9<br>A10	Is there difficulty feeding or drinking?<br>If yes, not able to drink of feed ANYTHING?	(1) Yes (2) No<br>(1) Yes (2) No	A31	Danger sign:<br>Not able to eat/drink anything?	(1) Yes (2) No
A11<br>A12	Is there vomiting?<br>If yes, vomits everything?	(1) Yes (2) No<br>(1) Yes (2) No	A32	Danger sign:<br>Vomits everything?	(1) Yes (2) No
A13<br>A14<br>A15	Are there red eyes?<br>If yes, for how long? ______ days<br>Presence of body rashes	(1) Yes (2) No<br><br>(1) Yes (2) No	A33<br>A34<br>A35	Danger sign:<br>Red eye for 4 days or more?<br>Red eye with body rashes<br>Sick but no danger sign:<br>Red eye less than 4 days?	(1) Yes (2) No<br>(1) Yes (2) No<br>(1) Yes (2) No
A16	Is there chest indrawing?	(1) Yes (2) No	A36	Danger sign: Any chest indrawing?	(1) Yes (2) No
A17<br>A18	If cough, how many breaths per minute? ______bpm<br>Is there fast breathing	(1) Yes (2) No<br>(1) Yes (2) No	A37	Sick but no danger sign:<br>Any fast breathing?	(1) Yes (2) No
A19	Is the child very sleep or unconscious?	(1) Yes (2) No	A38	Danger sign:<br>Very sleepy or unconscious?	(1) Yes (2) No
A20	Is there palmar pallor?	(1) Yes (2) No	A39	Danger sign: palmar pallor?	(1) Yes (2) No
A21	For child 6 months to 5 years, in which row does the child fall in weight in the weight for age chart?	(1) Yes (2) No	A40	Danger sign:	(1) Yes (2) No
A22	Is there swelling of both feet?	(1) Yes (2) No	A41	Danger sign:<br>Swelling on both feet?	(1) Yes (2) No

A23	Any other problem the CHA cannot treat?<br>Specify: ____________________	(1) Yes (2) No	A42	Other problem but no danger sign: Problem that CHA cannot treat?	(1) Yes (2) No

Any other danger sign or problem that the CHA cannot treat? (1) Yes (2) No

CHECK THE CHILD'S IMMUNIZATION STATUS

If the child is not being referred immediately, ask the caretaker for the child's vaccination card if available. If the child is being referred, skip this session and go to classifications.

B1. Does the caretaker have the child's vaccination card?
(1) Yes, and examiner sees card
(2) Yes, but card not available/provided to examiner
(3) No, does not have a card

If no the examiner should probe the caretaker to obtain as much information as possible regarding the chil's vaccines and record answers in question 2.

B2. Circle the vaccines received and circle immunizations still needed.

Birth	BCG	OPV-0
6 weeks	DPT-Hib + HepB 1	OPV-1
10 weeks	DPT-Hib + HepB 2	OPV-2
14 weeks	DPT – Hib + HepB 3	OPV-3
9 months	Measles	

B3. Ask the caretaker, did the HAS refer your child to receive a vaccination?
(A) Yes → If yes, which vaccines: ____
➔ If yes, when? ____
➔ If yes, where? ____
(B) No

B4. Evaluator, based on your re-examination and interview, answer the following questions:

(A) Should the case be managed at home? (1) Yes (2) No

(B) Should the case be referred to a health facility? (2) Yes (2) No

➔ If yes, which facility: ___________________________ (specify)

(C) If yes to A, what treatment should be prescribed to the child?*(Circle correct response below)*

	Treatment	Circle	
**A**	ORS	(1) Yes	(2) No
**B**	Zinc supplement	(3) Yes	(4) No
**C**	Paracetamol	(5) Yes	(6) No
**D**	Cotrimoxazole	(7) Yes	(8) No
**E**	Amoxycillin	(9) Yes	(10) No
**F**	Alu	(11) Yes	(12) No
**G**	Other	(13) Yes	(14) No

(D) Provide the following information about the treatment that should be provided

**A1**	Name			**B1**	Name	
**A2**	Formulation (i.e. tablet, sachet, liquid)			**B2**	Formulation	
**A3**	Amount each time			**B3**	Amount each time	
**A4**	Frequency			**B4**	Frequency	
**A5**	Total days			**B5**	Total days	
**A1**	Name			**B1**	Name	
**A2**	Formulation			**B2**	Formulation	
**A3**	Amount each time			**B3**	Amount each time	
**A4**	Frequency			**B4**	Frequency	
**A5**	Total days			**B5**	Total days	

(E) Does the child need a vaccination?

(1) Yes

➔ If yes, which vaccinations: ____

(2) No
