# Supplementary material for: Quality of Sick Child-Care Delivered by Community Health Workers in Tanzania
Source: Int J Health Policy Manag. 2018 Aug 15;7(12):1097–109. doi: 10.15171/ijhpm.2018.63 (PMC6358652; doi:10.15171/ijhpm.2018.63)
Supplement: Supplementary file 3 — Caretaker Exit Interview. [file ijhpm-7-1097-s003.pdf]

Connect Project: Quality Assessment of Community Health Agents Services

Form 4: Exit Interview – Caretaker of child (2 months – 5 years)

Date		District ID		Time Start	
Evaluator ID		CHA ID		Time End	
Child No					
Child Sex	M / F	Caretaker Sex	M / F		
Child Age (months)		Caretaker Relationship to child			

CARETAKER SATISFACTION

3.1 At the beginning of your interaction with the CHA, were you given the opportunity to express the state of health or symptoms of your child?

(1) Yes
(2) No
(88) Doesn't know
(99) No response

3.2 During your visit today, did the CHA ensure that the visit was done in privacy?

(1) Yes
(2) No
(88) Doesn't know
(99) No response

3.3 During the consultation were you given the opportunity to ask questions or express concerns about the investigations, the health problem and/or the treatment?

(1) Yes → continue to 3.4
(2) No → skip to 3.5
(88) Doesn't know → skip to 3.5
(99) No response → skip to 3.5

3.4 Did the CHA listen to your questions and concerns and give you satisfactory answers?
(1) Yes
(2) No
(7) NA
(88) Doesn't know
(99) No response

3.5 Overall, what is your opinion about the services you received from the CHA? Was it… *(read aloud)*
(1) Excellent
(2) Good
(3) Needs improvement – ***if yes ask*** how ____
____
____
____
(88) Doesn't know
(99) No response

3.6 The next time your child is sick, will you choose to see the CHA?

(1) Yes (skip to 3.8)
(2) No → *If no, ask:* Why not?

____

____

____ (ask 3.7)

(88) Doesn't know (ask 3.7)
(99) No response (ask 3.7)

3.7 The next time your child is sick where will you choose to seek care? *Probe for type of place (i.e. dispensary, health center, hospital) and name (i.e. Kibiti Health Center, etc.)*

(1) Dispensary __________________ (write name)
(2) Health center, ________________ (write name)
(3) Hospital _____________________ (write name)

(77) NA
(88) Doesn't know → Skip to 3.9
(99) No response → Skip to 3.9

3.8 Why will you choose…

→ *If answer to 3.6 is yes then ask,* **to have the CHA care for your sick child?**

➔ *If answer to 3.6 is no then ask,* **to seek care from (NAME OF PLACE GIVEN IN 3.7)?**

Circle all answers given:

(a) Distance
(b) Cost
(c) Time
(d) Perceived quality of services
(e) Attitude of provider
(f) Availability of desired treatment/services
(g) Other ____(specify)

(77) NA
(88) Doesn't know
(99) No response

CARETAKER COSTS

3.9 From the time when you/someone from the household *decided to seek care from CHA*, how long do you feel it took until your child was attended by the CHA?

Write

__________ **[days]** __________ **[hours]** __________ **[minutes]**

If respondent cannot recall time in terms of days, hours or minutes then read all of the following responses, and ask respondent to provide ONE answer:

(1) A very long time
(2) Long time
(3) Acceptable time
(4) Quickly
(5) Very quickly

(88) Doesn't know
(99) No response

4.1 Did you need to use any form of transport in order to obtain care from the CHA?
(1) Yes, *if yes then ask*, **what type of transport?** ____________________________(specify).
(2) No
(88) Doesn't know
(99) No response

4.2 Did you have to spend any money or pay anything in order to obtain care from the CHA?
(1) Yes, *if yes then ask*, **how much?** ____________________________(specify).
(2) No
(88) Doesn't know
(99) No response

4.3 Before obtaining care from the CHA, did you seek help or care from anyone else for your sick child?
(1) Yes
(2) No → skip to 4.6
(88) Doesn't know → skip to 4.6
(99) No response → skip to 4.6

4.4 Did you seek help from any of the following:

A. Traditional healer
(1) Yes
(2) No → Skip to B
a) How much did the services cost you? __________ TSH (888) DK
b) How much did it cost you to travel for these services? __________ TSH (888) DK

B. Local shop
(1) Yes
(2) No → Skip to B
a) How much did the services cost you? __________ TSH (888) DK
b) How much did it cost you to travel for these services? __________ TSH (888) DK

C. Drug Vendor
(1) Yes
(2) No → Skip to B
a) How much did the services cost you? __________ TSH (888) DK
b) How much did it cost you to travel for these services? __________ TSH (888) DK

D. Other: __________________(specify)
(1) Yes
(2) No → Skip to B
a) How much did the services cost you? __________ TSH (888) DK
b) How much did it cost you to travel for these services? __________ TSH (888) DK

(77) NA
(88) Doesn't know
(99) No response

4.5 Before starting to seek services from the CHA, how much time do you feel you spent seeking care from (NAME PLACES WHERE RESPONDENT SOUGHT CARE GIVEN IN 3.10)?
(1) 1 day or less
(2) 2-3 days

(3) 3-7 days
(4) More than 7 days
(77) NA
(88) Doesn't know
(99) No response

4.6 From the time you first noticed that your child was sick, how much time passed before you started to seek care from….

→ If 4.3 in 'no' then say: **CHA?**
➔ If 4.3 is 'yes' then say **[names of places given in 4.4]?**

(1) 1 day or less
(2) 2-3 days
(3) 3-7 days
(4) More than 7 days
(7) NA
(77) NA
(88) Doesn't know
(99) No response

4.7 How many days have you or other family members spent taking care of your child instead of working, including time spent with the CHA today?

__________________ (write number of days).

(88) Doesn't know
(99) No response

4.8 How much income have you or other family members lost as a result of taking care of your child instead of working?

________________ (write TSH)

(88) Doesn't know
(99) No response

MEDICATIONS PRESCRIBED AND UNDERSTANDING

4.9 Did the CHA give you or prescribe any medicines for your child today?

→ *By medicine it means Alu, cotrimoxazole, amoxicillin, paracetamol and zinc. See #3.20 for ORS*
➔ *Medicines prescribed include only those received by caretaker from CHA, not those available at referral point.*

(1) Yes → compare caretaker's medication with the samples for identification of medicines.
(2) No → skip to
(88) Doesn't know → skip to 5.5
(99) No response

5.0 What medicine was prescribed or given?

→ *Copy the information form the caretaker's medication or prescription:*

(a) Name: ___________________________________
(b) Formulation: _______________________________

➔ Then ask caretaker *(record what you hear):*
(c) How much will you give your child each time: ___________________________ (888 = don't know).
(d) How many times will you give it to your child each day? ___________________ (888 = don't know).
(e) How many days will you give the medicine to your child? ______________days (888 = don't know).

(77) NA
(89) Doesn't know
(99) No response

5.1 Did the CHA prescribe a SECOND medicine? (1) Yes (2) No → Skip to 5.5
→ *Copy the information form the caretaker's medication or prescription:*
(a) Name: ___________________________________
(b) Formulation: _______________________________

➔ Then ask caretaker *(record what you hear):*
(c) How much will you give your child each time: ___________________________ (888 = don't know).
(d) How many times will you give it to your child each day? ___________________ (888 = don't know).
(e) How many days will you give the medicine to your child? ______________days (888 = don't know).

(77) NA → Skip to 5.5
(90) Doesn't know → Skip to 5.5
(99) No response → Skip to 5.5

5.2 Did the CHA prescribe a THIRD medicine? (1) Yes (2) No → Skip to 5.5
→ *Copy the information form the caretaker's medication or prescription:*
(a) Name: ___________________________________
(b) Formulation: _______________________________

➔ Then ask caretaker *(record what you hear):*
(c) How much will you give your child each time: ___________________________ (888 = don't know).
(d) How many times will you give it to your child each day? ___________________ (888 = don't know).
(e) How many days will you give the medicine to your child? ______________days (888 = don't know).

(77) NA → Skip to 5.5
(88) Doesn't know → Skip to 5.5
(99) No response → Skip to 5.5

5.3 Did the CHA prescribe a FOURTH medicine? (1) Yes (2) No → Skip to 5.5
→ *Copy the information form the caretaker's medication or prescription:*
(a) Name: ___________________________________
(b) Formulation: _______________________________

➔ Then ask caretaker *(record what you hear):*
(c) How much will you give your child each time: ___________________________ (888 = don't know).
(d) How many times will you give it to your child each day? ___________________ (888 = don't know).
(e) How many days will you give the medicine to your child? ______________days (888 = don't know).

(77) NA → Skip to 5.5
(89) Doesn't know → Skip to 5.5
(99) No response → Skip to 5.5

5.4 Did the CHA prescribe a FIFTH medicine? (1) Yes (2) No → Skip to 5.5
→ *Copy the information form the caretaker's medication or prescription:*

Form 4: Exit Interview – Caretaker of child (2 months – 5 years)

(a) Name: ___________________________________
(b) Formulation: _______________________________

➔ Then ask caretaker *(record what you hear):*
(c) How much will you give your child each time: ___________________________ (888 = don't know).
(d) How many times will you give it to your child each day? ___________________ (888 = don't know).
(e) How many days will you give the medicine to your child? ______________days (888 = don't know).

(77) NA → Skip to 5.5
(90) Doesn't know → Skip to 5.5
(99) No response → Skip to 5.5

5.5 Was ORS prescribed or given?
(1) Yes
(2) No → skip to 5.6

5.5.a. How much water will you mix with one ORS packet? ________________ (888 = don't know)
5.5.b When will you give ORS to you child each day? ___________________ (888 = don't know)
5.5.c How much ORS will you give to your child each time? _______________(888 = don't know)

5.6 Did the CHA make a follow up appointment with you?
(1) Yes → then ask, in how many days? __________days (888 = don't know)
(2) No
(8) Doesn't know

5.7 Sometimes a child's condition may worsen and he/she should be taken immediately to a health facility: what types of symptoms would cause you to take your child to a health facility right away? *(Circle all that respondent says. Do not prompt, but keep asking for more until caretaker cannot recall any additional ones).*

A. Child not able to feed or drink. (1) Mentioned (2) Not mentioned
B. Child becomes sicker. (1) Mentioned (2) Not mentioned
C. Child has blood in the stools. (1) Mentioned (2) Not mentioned
D. Other, _________________________
_________________________*(specify)*_
