# Supplementary material for: Quality of Sick Child-Care Delivered by Community Health Workers in Tanzania
Source: Int J Health Policy Manag. 2018 Aug 15;7(12):1097–109. doi: 10.15171/ijhpm.2018.63 (PMC6358652; doi:10.15171/ijhpm.2018.63)
Supplement: Supplementary file 4 — WAJA Support Questionnaire. [file ijhpm-7-1097-s004.pdf]

Connect Project: Quality Assessment of Community Health Agents Services

Form 8: Supply and Support Checklist

Date		District ID		Time Start	
Evaluator ID		CHA ID		Time End	

EQUIPMENT AND SUPPLIES

→ *Ask the CHA to show you around where he or she works.* ***Look and touch*** *to complete the following questions.*

8.1 Does the CHA have the following equipment and materials?

1. Watch or timing advice
2. Supplies to mix ORS, cups, spoons
3. MUAC tape
4. Medicine stock forms
5. Referral forms
6. Service delivery register
7. Working bicycle
8. Bangu Kitita (job aid for IMCI)
9. Malaria RDT
10. Gauze
11. Cotton wool
12. Scissors
13. Thermometer

14 Other materials: ____

8.2 Are the drugs and supplies in the lock box? (1) Yes (2) No

8.3 Does the CHA have ready access to the lock box? (1) Yes (2) No

8.4 Who holds the other key to the lock box? ______________________ (write name)

AVAILABILITY OF DRUGS

➔ *Check the drug stocks. Answer the following questions based on what you* ***see****.*

8.5 Does the CHA have the following drugs available the day of the visit?

A. ORS
B. Cotrimoxazole
C. Alu
D. Paracetamol
E. Zinc tablets
F. Oral contraceptives
G. Condoms
H. Mebendazole
I. Iodine tincture
J. Methylated spirit

➔ *Ask the following questions to the CHA. Use any documentation (e.g. service delivery register for information about dispending drugs, monthly summary forms).*

8.6 Have you experienced a stock out in the last three months of any of the following? If yes, for about how many days did you not have that drug or supply?

Form 8: Supply and Support Checklist

A	ORS	(1) Yes (2) No (3) Never received	Ai	Duration of stock out: ______ days.
B	Cotrimoxazole	(1) Yes (2) No (3) Never received	Bi	Duration of stock out: ______ days.
C	Alu	(1) Yes (2) No (3) Never received	Ci	Duration of stock out: ______ days.
D	Paracetamol	(1) Yes (2) No (3) Never received	Ci	Duration of stock out: ______ days.
E	Zinc tablets	(1) Yes (2) No (3) Never received	Ci	Duration of stock out: ______ days.
F	Oral contraceptives	(1) Yes (2) No (3) Never received	Ci	Duration of stock out: ______ days.
G	Condoms	(1) Yes (2) No (3) Never received	Ci	Duration of stock out: ______ days.
H	Mebendazole	(1) Yes (2) No (3) Never received	Ci	Duration of stock out: ______ days.
I	Iodine tincture	(1) Yes (2) No (3) Never received	Ci	Duration of stock out: ______ days.
J	Methylated spirit	(1) Yes (2) No (3) Never received	Ci	Duration of stock out: ______ days.

CHA SERVICES

8.7 Which of the following services have you performed in the community in the past 4 weeks?
(Read the list to CHA)

A. Community case management of sick children
B. Outreach (with facility counterparts) → if yes specify which: ________________________________ ____ (e.g. vaccination, ANC)
C. Environmental health (water and sanitation)
D. Growth monitoring/nutritional activities
E. HIV/VCT
F. Family planning
G. Safe motherhood counseling
H. Newborn care counseling
I. Group health education ____
J. Planning, meeting with village authorities ____
K. Other: ______________________________
L. Other: ______________________________
M. Other: ______________________________

8.8. For each service listed above, where in the community do you provide the service?

A. Community case management of sick children: ________________________ (name of place)
B. Outreach: ____
C. Environmental health (water and sanitation) ____
D. Growth monitoring/nutritional activities____
E. HIV/VCT ____
F. Family planning ____
G. Safe motherhood counseling ____
H. Newborn care counseling ____
I. Group health education ____
J. Planning, meeting with village authorities ____

K Other: ______________________________
L. Other: ______________________________
M: Other: ______________________________

8.9 During a typical week about what percentage of your time do you feel you spend doing each of those activities?

A.	Community case management of sick children	___%
B.	Outreach (with facility counterparts)	___%
C.	Environmental health (water and sanitation)	___%
D.	Growth monitoring/nutritional activities	___%
E.	HIV/VCT	___%
F.	Family planning	___%
G.	Safe motherhood counseling	___%
H.	Newborn care counseling	___%
I.	Group health education	___%
J.	Planning, meeting with village authorities	___%
I	Paperwork and completing registers	___%
I.	Other: ______________________	___%
J.	Other: ______________________	___%
K:	Other: ______________________	___%

CHA SUPERVISION AND SUPPORT

Review the monthly summary reports for the past three months and write down number of times in past three months the CHA received:

8.10 Supervision from health facility supervisor: ______ times

8.11 Supervision from community supervisor: ______ times

8.12 How many times during the last three months did you receive a supervisory visit specific to (read aloud all options and write down number)…

A.	Case management of sick children	______ times
B.	Other IMCI	______ times
C.	Family planning	______ times
D.	HIV prevention/other Reproductive Health	______ times
E.	Safe motherhood promotion	______ times
F.	Essential newborn care	______ times

Refer to monthly summary forms and write the month of the most recent visit from health facility supervisor: _______

8.13 At the MOST RECENT supervision visit from your HEALTH FACILITY SUPERVISOR, did the supervisor do any of the following? *(Read list to CHA)*

A.	Deliver any supplies	(1) Yes	(2) No	(8) DK
B.	Check your records or reports	(1) Yes	(2) No	(8) DK
C.	Observe any of your work	(1) Yes	(2) No	(8) DK
D.	Observe you managing a sick child	(1) Yes	(2) No	(8) DK
E.	Observe you counseling a pregnant woman	(1) Yes	(2) No	(8) DK
F.	Observe you providing family planning services	(1) Yes	(2) No	(8) DK
G.	Observe performing other IMCI tasks	(1) Yes	(2) No	(8) DK

H.	Use a supervision checklist	(1) Yes	(2) No	(8) DK
I.	Provide positive feedback	(1) Yes	(2) No	(8) DK
J.	Correct you or remind you of things you've forgotten	(1) Yes	(2) No	(8) DK
K.	Provide updates on administrative or technical issues	(1) Yes	(2) No	(8) DK
L.	Discuss problems you have encountered	(1) Yes	(2) No	(8) DK
M.	Answered questions about your work	(1) Yes	(2) No	(8) DK

Refer to monthly summary forms and write the month of the second most recent visit from health facility supervisor: _______

8.14 At the SECOND MOST RECENT supervision visit from your HEALTH FACILITY SUPERVISOR, did the supervisor do any of the following? *(Read list to CHA)*

A.	Deliver any supplies	(1) Yes	(2) No	(8) DK
B.	Check your records or reports	(1) Yes	(2) No	(8) DK
C.	Observe any of your work	(1) Yes	(2) No	(8) DK
D.	Observe you managing a sick child	(1) Yes	(2) No	(8) DK
E.	Observe you counseling a pregnant woman	(1) Yes	(2) No	(8) DK
F.	Observe you providing family planning services	(1) Yes	(2) No	(8) DK
G.	Observe performing other IMCI tasks	(1) Yes	(2) No	(8) DK
H.	Use a supervision checklist	(1) Yes	(2) No	(8) DK
I.	Provide positive feedback	(1) Yes	(2) No	(8) DK
J.	Correct you or remind you of things you've forgotten	(1) Yes	(2) No	(8) DK
K.	Provide updates on administrative or technical issues	(1) Yes	(2) No	(8) DK
L.	Discuss problems you have encountered	(1) Yes	(2) No	(8) DK
M.	Answered questions about your work	(1) Yes	(2) No	(8) DK

Refer to monthly summary forms and write the month of the third most recent visit from health facility supervisor: _______

8.15 At the THIRD MOST RECENT supervision visit from your HEALTH FACILITY SUPERVISOR, did the supervisor do any of the following? *(Read list to CHA)*

A.	Deliver any supplies	(1) Yes	(2) No	(8) DK
B.	Check your records or reports	(1) Yes	(2) No	(8) DK
C.	Observe any of your work	(1) Yes	(2) No	(8) DK
D.	Observe you managing a sick child	(1) Yes	(2) No	(8) DK
E.	Observe you counseling a pregnant woman	(1) Yes	(2) No	(8) DK
F.	Observe you providing family planning services	(1) Yes	(2) No	(8) DK
G.	Observe performing other IMCI tasks	(1) Yes	(2) No	(8) DK
H.	Use a supervision checklist	(1) Yes	(2) No	(8) DK
I.	Provide positive feedback	(1) Yes	(2) No	(8) DK
J.	Correct you or remind you of things you've forgotten	(1) Yes	(2) No	(8) DK
K.	Provide updates on administrative or technical issues	(1) Yes	(2) No	(8) DK
L.	Discuss problems you have encountered	(1) Yes	(2) No	(8) DK
M.	Answered questions about your work	(1) Yes	(2) No	(8) DK

8.16 During supervisions from your HEALTH FACILITY SUPERVISOR would you say that the service delivery information from your registers and reports are used [read options aloud]…

1. Always
2. Sometimes
3. Rarely
4. Never

Form 8: Supply and Support Checklist

Refer to monthly summary forms and write the month of the most recent supervision from village supervisor: _______

8.17 At the MOST RECENT supervision visit from your VILLAGE SUPERVISOR, did the supervisor do any of the following? *(Read list to CHA)*

A.	Organize or review work plan	(1) Yes	(2) No	(8) DK
B.	Plan services outreach activities	(1) Yes	(2) No	(8) DK
	→ If yes, what was the topic: _______________________			
C.	Review register	(1) Yes	(2) No	(8) DK
D.	Make household visits	(1) Yes	(2) No	(8) DK
E.	Use supervision checklist	(1) Yes	(2) No	(8) DK
F.	Provide positive feedback	(1) Yes	(2) No	(8) DK
G.	Correct you or remind you of things you've forgotten	(1) Yes	(2) No	(8) DK
H.	Provide updates on important community issues	(1) Yes	(2) No	(8) DK
I.	Discuss problems you have encountered in the village	(1) Yes	(2) No	(8) DK
J.	Resolve and disputes or problems with you in the village	(1) Yes	(2) No	(8) DK
K.	Answered questions about your work in the community	(1) Yes	(2) No	(8) DK
E.	Other: ____ (specify)			

Refer to monthly summary forms and write the month of the second most recent supervision from village supervisor: _______

8.18 At the SECOND MOST RECENT supervision visit from your VILLAGE SUPERVISOR, did the supervisor do any of the following? *(Read list to CHA)*

A.	Organize or review work plan	(1) Yes	(2) No	(8) DK
B.	Plan services outreach activities	(1) Yes	(2) No	(8) DK
	→ If yes, what was the topic: _______________________			
C.	Review register	(1) Yes	(2) No	(8) DK
D.	Make household visits	(1) Yes	(2) No	(8) DK
E.	Use supervision checklist	(1) Yes	(2) No	(8) DK
F.	Provide positive feedback	(1) Yes	(2) No	(8) DK
G.	Correct you or remind you of things you've forgotten	(1) Yes	(2) No	(8) DK
H.	Provide updates on important community issues	(1) Yes	(2) No	(8) DK
I.	Discuss problems you have encountered in the village	(1) Yes	(2) No	(8) DK
J.	Resolve and disputes or problems with you in the village	(1) Yes	(2) No	(8) DK
K.	Answered questions about your work in the community	(1) Yes	(2) No	(8) DK
E.	Other: ____ (specify)			

Refer to monthly summary forms and write the month of the third most recent supervision from village supervisor: _______

8.19 At the THIRD MOST RECENT supervision visit from your VILLAGE SUPERVISOR, did the supervisor do any of the following? *(Read list to CHA)*

A.	Organize or review work plan	(1) Yes	(2) No	(8) DK
B.	Plan services outreach activities	(1) Yes	(2) No	(8) DK
	→ If yes, what was the topic: _______________________			
C.	Review register	(1) Yes	(2) No	(8) DK
D.	Make household visits	(1) Yes	(2) No	(8) DK
E.	Use supervision checklist	(1) Yes	(2) No	(8) DK
F.	Provide positive feedback	(1) Yes	(2) No	(8) DK
G.	Correct you or remind you of things you've forgotten	(1) Yes	(2) No	(8) DK
H.	Provide updates on important community issues	(1) Yes	(2) No	(8) DK
I.	Discuss problems you have encountered in the village	(1) Yes	(2) No	(8) DK

J. Resolve and disputes or problems with you in the village (1) Yes (2) No (8) DK
K. Answered questions about your work in the community (1) Yes (2) No (8) DK
E. Other: ____ (specify)

8.20 During supervisions from your VILLAGE SUPERVISOR would you say that the service delivery information from your registers and reports are used [read options aloud]…

1. Always
2. Sometimes
3. Rarely
4. Never

CHA RELATIONSHIP WITH VILLAGE STAKEHOLDERS AND GOVERNMENT

Review the activities that were mentioned by the CHA in 8.7, 8.8 and 8.9

8.21 For [READ ALOUD ACTIVITIES MENTIONED IN 8.1, 8.8 AND 8.9), who are the stakeholders that you work with in order to complete the work? *(write all stakeholders you hear for each activity)*

A. Community case management of sick children: ____________________________________
B. Outreach: ____
C. Environmental health (water and sanitation) ____________________________________
D. Growth monitoring/nutritional activities____________________________________
E. HIV/VCT ____
F. Family planning ____
G. Safe motherhood counseling ____
H. Newborn care counseling ____
I. Group health education ____
J. Planning, meeting with village authorities ____________________________________
K Other: ____
L. Other: ____
M: Other: ____

List the six stakeholders most frequently cited in 8.23: ____________________________________
____
____

8.22 For [SAY EACH STAKEHOLDER WRITTEN ABOVE] in the past three months how many times have you met with her/him for work purposes:

(1) ________________________ (write name); _____ times
(2) ________________________ (write name); _____ times
(3) ________________________ (write name); _____ times
(4) ________________________ (write name); _____ times
(5) ________________________ (write name); _____ times

8.23 Since you started your job as CHA would you say that you have met with members of the village government… *(read all options aloud)*

(1) Frequently
(2) Sometimes
(3) Rarely
(4) Never → skip to 8.25

8.24 What did you do when you met with members of the village government? Did you… *(read each option aloud)?*

A. Report the activities that you have carried out? ___ Yes ___ No

B. Plan activities that you will carry out? ___ Yes ___ No
C. Collaborate together on implementing activities in the community? ___ Yes ___ No
D. Other things:____

8.25 Since you started your job as CHA would you say that you have reported to the community decisions or commitments taken on by your village government…

(1) Frequently
(2) Sometimes
(3) Rarely
(4) Never

8.26 Since you started your job as CHA, would you say that you have met with met with the health facility governance committee to plan community health activities…

(1) Frequently
(2) Sometimes
(3) Rarely
(4) Never → 8.28

8.27 What did you do when you met with members of the health facility governance committee? Did you… *(read each option aloud)?*

A. Report the activities that you have carried out? ___ Yes ___ No
B. Plan activities that you will carry out? ___ Yes ___ No
C. Collaborate together on implementing activities in the community? ___ Yes ___ No
D. Other things: ____

8.28 Since you started your job as CHA would you say that you have reported to the community decisions or commitments taken on by your health facility governance committee…

(1) Frequently
(2) Sometimes
(3) Rarely
(4) Never

8.29 In the past three months how many community health activities have you carried out?

(1) Yes → Number of activities _______

8.30 What community health activities did you plan? *(Write activities)*

____
____
____

8.31 Who did you work with to hold those activities? Did you work with *(read all options aloud)…*

A. Village supervisor ___ Yes ___ No
B. Village health committee ___ Yes ___ No
C. Health facility supervisor ___ Yes ___ No
D. Health facility governing committee ___ Yes ___ No
E. Members of CHMT ___ Yes ___ No
F. Members of the Connect Project ___ Yes ___ No
G. Other: ____

8.32 Since you started your job as CHA would you say that you have met with your health facility governance committee to report what you have done related to you job…

(1) Frequently
(2) Sometimes
(3) Rarely
(4) Never

CHA PLANNING, MONITORING AND INFORMATION USE

8.30 Since you started your job as CHA, how many times have you conducted a rapid assessment in your community?
(1) Never → 8.35
(2) ____ times
(8) Don't know

8.31 Who did you work with to complete the rapid assessments?
(1) ___________________________ (write name);
(2) ___________________________ (write name);
(3) ___________________________ (write name);
(4) ___________________________ (write name);
(5) ___________________________ (write name);

8.32 What steps did you take to complete the assessments? *(Listen and circle all the things CHA mention).*
A.
B.
C.
D.
E.
F.

8.33 *Ask to see where and how the findings from the rapid assessments are kept and managed?*
(1) Findings available and seen by interviewer
(2) Findings reported to be available, but not seen
(3) Findings not available
(4) Findings never documented

8.34 How do you use the information you obtain from the rapid assessment? *(Listen and circle all the things CHA mention – if necessary probe for 'planning at the community level').*
A.
B.
C.
D.
E.
F.

8.35 What steps did you use to complete to plan your work in your community? *(Listen and circle all the things CHA mention).*
A.
B.
C.
D.

E.
F.
G. I haven't prepared a work plan in my community. → skip to 8.40

8.36 *Ask to see the current CHA work plan.*
(1) Work plan available and seen by interviewer
(2) Work plan reported to be available, but not seen
(3) Work plan not available
(4) Work plan never documented

8.37 After completing your plans do you take steps to monitor the implementation of your plan?
(1) Yes
(2) No → skip to 8.40
(8) Don't know

8.38 What do you do to monitor your work plan? *(Listen and circle all the things CHA mention).*
A.
B.
C.
D.
E.
F.

8.39 *Ask to see the current monitoring and evaluation tools.*
(1) Tools available and seen by interviewer
(2) Tools plan reported to be available, but not seen
(3) Tools plan not available
(4) Tools plan never documented

CHA RECORDS

8.40 Review the CHA service delivery register and calculate the following using the past 1 year of information from the register (for CHA batch 1) OR all information from the register (for CHA batch 2):

DO NOT COUNT MULTIPLE VISITS FOR THE SAME CLIENT – RECORD NUMBER CLIENTS IN EACH CATEGORY

A. The number of women CHA provided oral contraceptives __________
B. The number of pregnant women that the CHA visited once __________
C. The number of newborns/postpartum mothers the CHA visited once: __________
D. The number of sick children the CHA attended __________

8.41 Refer to all the FAMILY PLANNING CLIENTS of the CHA [from 8.33A]
A. The number of women that received follow up from CHA within 2 weeks of receiving OC from CHA __________
B. The number of women that received a follow up from CHA within 2-3 weeks __________
C. The number of women received a follow up from the CHA within 3-4 weeks __________
D. The number of women that never received a follow up from the CHA __________

8.42 Refer to all the PREGNANT WOMEN attended by CHA [from 8.33B]
(1) Number that received 4 visits from CHA

(2) Number that received 3 visits from CHA
(3) Number that received 2 visits from CHA
(4) Number that received 1 visit from CHA

(5) Health center, ________________ (write name)
(6) Hospital _____________________ (write name)

8.43 Refer to all the NEWBORNS/POSTPARTUM MOTHERS attended by CHA [from 8.33C]

(1) Number that received 4 visits from CHA
(2) Number that received 3 visits from CHA
(3) Number that received 2 visits from CHA
(4) Number that received 1 visit from CHA

8.44 Refer to all the sick children that were attended by CHA [from 8.33C]

(1) Number that received a follow up from CHA within 1-3 days of the first sick child visit _____
(2) Number that received a follow up from CHA within 4-7days of the first sick child visit _____
(3) Number that received a follow up from CHA within 8-10 days of the first sick child visit _____
(4) Number that received a follow up from the CHA within 11-14 days of the first sick child visit _____
(5) The number that never received a follow up from the CHA _____
